# Epilepsy awareness among physicians in plains and plateau regions of western China: a cross-sectional study

**DOI:** 10.1186/s42494-026-00251-9

**Published:** 2026-03-11

**Authors:** Xi Xiao, Jing Xiao, Changqiong Zhou, Chunge Pan, Yu Hao, Minghao Jin, Mingzhe Han, Shuang Jiang, Jiawei Liu, Yingying Tang, Xintong Wu, Bo Yan, Xiaoting Hao

**Affiliations:** 1 Department of Neurology, West China Hospital, Sichuan University, Chengdu, 610041 China; 2 West China School of Public Health and West China Fourth Hospital, Sichuan University, Chengdu, 610041 China; 3 Liangshan Hospital of Integrated Traditional and Western Medicine, Liangshan Yi Autonomous Prefecture, Liangshan, 615050 China; 4 Department of Neurology, People’s Hospital of Tibet Autonomous Region, Lhasa, 850000 China; 5 No. 37 Guoxue Alley, Chengdu, Sichuan Province 610000 China

**Keywords:** Epilepsy, Medical knowledge, Regional disparities, Cross-sectional study

## Abstract

**Background:**

Epilepsy is a common neurological disorder affecting about 50 million people worldwide. Disparities in healthcare resources lead to geographical variation in its diagnosis, treatment, and management. In particular, the gap between plains and plateau regions in western China remains understudied. Assessing healthcare professionals’ expertise and delivering targeted training represent effective strategies to narrow this treatment gap. We compare epilepsy knowledge between physicians in more developed plains regions and less developed plateau regions of western China in order to inform targeted interventions to address regional disparities.

**Methods:**

In this cross-sectional study, physicians attending epilepsy training sessions in three cities representative of the western plains region of China (Chengdu) and of the western plateau region (Lhasa and Xichang) completed a questionnaire assessing knowledge in several modules of the disease, including its diagnosis, preoperative evaluation, drug and surgical treatments, and management during pregnancy.

**Results:**

Of the 349 participants given questionnaires, 325 (93.1%) were included in the final analysis. Physicians from the plateau region scored significantly lower than physicians from the plains region on knowledge about epilepsy diagnosis (Number of questions with discrepancies / Total number of questions in this module, i.e., 7/7, *P* < 0.05), definition of drug-resistant epilepsy (2/2 questions *P* < 0.05), preoperative evaluation and surgical treatments (7/14 questions *P* < 0.05), status epilepticus (2/2 questions *P* < 0.05), epilepsy comorbidities (2/2 questions *P* < 0.05), and epilepsy management during pregnancy (3/3 questions *P* < 0.05). However, no significant differences were found between neurology specialists in plateau regions and those in plain regions across all six epilepsy knowledge modules and the overall score (0.542 < *P* < 0.829).

**Conclusions:**

Significant gaps persist in epilepsy care knowledge among physicians in western plateau of China, particularly in diagnosis, treatment, and specialized management. Both plateau and plains regions lack awareness of advanced preoperative evaluation techniques. Targeted training and capacity-building interventions are needed, with effectiveness evaluated through longitudinal studies.

**Graphical Abstract:**

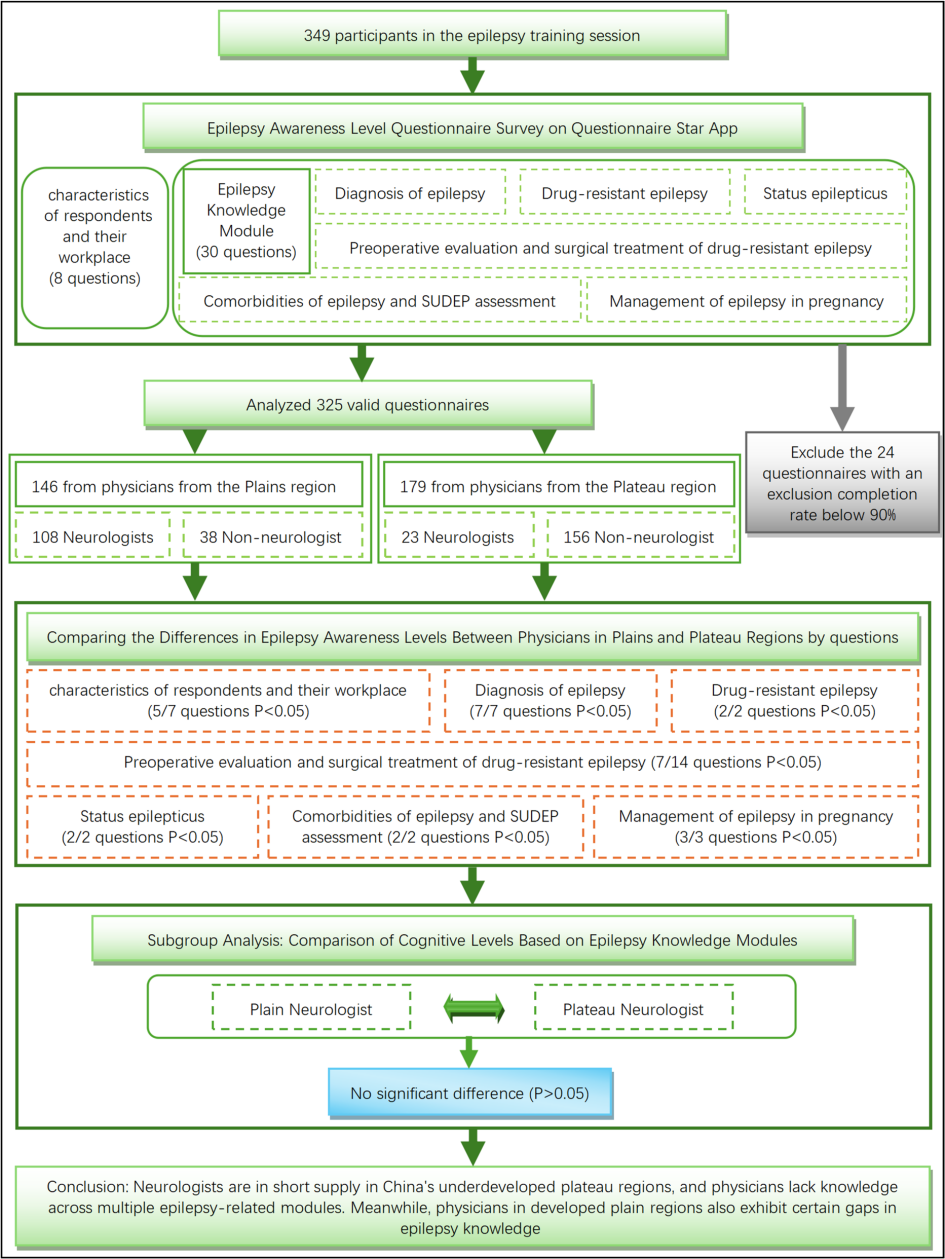

**Supplementary Information:**

The online version contains supplementary material available at 10.1186/s42494-026-00251-9.

## Background

Epilepsy affects approximately 50 million people worldwide [1], with an incidence of 61.4 (95% confidence interval, 39.0–61.1) per 100,000 person-years and overall lifetime prevalence of 7.60 per 1000 [2]. Epilepsy ranks seventh among neurological disorders in terms of disability-adjusted life years [3], which translates to substantial physical, mental and emotional burden for affected individuals and their families. In 2021, the disorder was responsible for approximately 140 deaths (95% confidence interval, 116–153 deaths) per 100,000 people [3].

In China's efforts to treat and manage the burden of epilepsy across the country, the country must deal with the challenge of healthcare disparities: medical infrastructure, resources and education are generally much more advanced in the more prosperous northern, eastern and southeastern regions than in the less developed western regions. For example, the number of physicians per 1,000 people is 1.22 in Tianjin, 1.86 in Jiangsu, and 1.28 in Hunan, but only 1.02 in Sichuan and 0.84 in Tibet [4]. Healthcare disparities are substantial even within western China: the plains area around Chengdu, the capital of Sichuan province, is relatively more developed and is the site of most epilepsy centers in western China [5]. In contrast, on the Tibetan Plateau farther west, nearly 90% of epilepsy patients do not receive effective treatment in large part due to lack of adequate healthcare resources, including properly trained staff [6]. More than 90% of epilepsy treatment and management occurs in tertiary hospitals, which are far less numerous in plateau regions than in plains regions [5].

The issue of resource constraints in epilepsy care is not merely a problem confined to certain regions of China; it represents a significant challenge faced by numerous low- and middle-income countries (LMICs) globally [7]. In Africa, for instance, there is a severe shortage of neurologists, with sub-Saharan Africa exhibiting one of the world's lowest neurologist densities. Additionally, the absence of specialized training and the limited expertise among primary care providers have been identified as major obstacles contributing to the epilepsy care gap in East Africa [8]. A study conducted in rural Bolivia demonstrated that continuous education campaigns targeting "lay health-care workers" in rural communities were highly effective. These initiatives not only significantly enhanced participants' knowledge of epilepsy but also played a crucial role in minimizing associated stigma and reducing the treatment gap [9].

The International League Against Epilepsy (ILAE) and the World Health Organization stress the importance of long-term, standardized management of epilepsy patients, which requires that medical staff be appropriately trained in numerous aspects of the disease, including its diagnosis [10–12], recognition and treatment of drug-resistant epilepsy [13–16], preoperative evaluation [13, 17, 18], recognition and treatment of status epilepticus [19], monitoring and management of comorbidities and risk of sudden death [20, 21], and management of epilepsy during pregnancy [22, 23]. The Chinese Association Against Epilepsy has initiated programs such as "The Going West Project" [24], which have been successful at increasing epilepsy awareness among medical staff in the west of the country [25]. However, the level of awareness among physicians in plateau regions regarding key aspects of this disease remains insufficient, particularly when compared to physicians in plain regions whose awareness has improved [26–29].

Here we undertook a cross-sectional survey to compare the level of knowledge between physicians in Chengdu plain and those in two cities in the plateau region on key aspects of epilepsy important for standardizing its treatment and management. The study also compared the epilepsy knowledge levels between the neurologists in plateau and plain regions to validate the significant role of specialized epilepsy training or education in improving epilepsy awareness. The comparative analysis provides a foundation for planning epilepsy training in the two regions and thereby reducing regional disparities in epilepsy care.

## Methods

### Research design and participants

In this cross-sectional study, physicians who were signing up for a training course on the diagnosis, long-term management, preoperative evaluation, and surgical treatment of epilepsy from March to April 2023 in Chengdu, Lhasa, and Xichang in China were invited to participate in our survey. Training participation is voluntary. Participants who provided informed consent were required to complete an online questionnaire (more details in Section "Questionnaire") before the training began. Physicians enrolled in the training were either currently engaged in or planning to engage in epilepsy diagnosis and treatment.

Respondents were assigned to either the "plains group" if they lived and worked in Chengdu, which lies at an altitude of 500 m on the Chengdu Plain; or the "plateau group" if they lived and worked in Lhasa or Xichang, which lie on the Qinghai-Tibet Plateau, whose height ranges from 1,500 to 5,000 m.

### Questionnaire

A questionnaire was designed specifically for this study, and it was based on consensus guidelines from the ILAE and the research literature [7–19, 21] (Supplementary Table). The first nine items collected general information about respondents and their workplace. The remaining 30 items asked respondents about their awareness or knowledge of key aspects of epilepsy diagnosis, treatment and management. The only correct response for questions 10–12 was "Additional information is needed to answer the question", which was awarded 1 point; all other responses to these questions were awarded 0 point. Responses to questions 13–39 were on a 5-point Likert scale, where 1= "Completely unaware/no knowledge", 2 = "Somewhat aware/little knowledge", 3 = "Aware/some knowledge", 4 = "Fairly aware/reasonable knowledge", and 5 = "Extremely aware/detailed knowledge". Total possible scores on the questionnaire ranged from 27 to 138.

The questionnaire was distributed to participants through the Questionnaire Star App (https://www.wjx.cn/).

### Data processing and analysis

Questionnaires on which fewer than 34 items (90%) contained responses were excluded from analysis. The remaining questionnaires were analyzed using SPSS 23.0 (IBM, Armonk, NY, USA). Categorical data were reported as N (Percentage) or Median (IQR). Differences in demographic and professional characteristics between respondents from plains or plateau regions were assessed for significance using the chi-squared test or Fisher's exact test, except that differences in professional rank were assessed using the Mann–Whitney U test. All analyses comparing differences in consciousness levels employed the Mann–Whitney U test.

## Results

Of the 349 questionnaires received from physicians working in various medical institutions, 24 were excluded because at least 5 of the 38 items (10%) did not have responses, leaving 325 (93.1%) in the final analysis. The proportion of female participants exceeded that of male participants in both regions, with 64.38% in the plains region and 67.04% in the plateau region. However, no significant difference was observed between the two regions. Approximately 90% of the 146 respondents from the plains region and of the 179 respondents from the plateau region worked in tertiary hospitals, with more than 93% of all respondents working in inpatient departments and a smaller proportion working also or only in outpatient departments (Table 1). Just over half in each group had been working in health care for 5–15 years. Just under half of plains staff worked at the level of specialist, whereas just over half of plateau staff were residents. A significantly higher proportion of plains staff than plateau staff specialized in neurology (73.97% vs. 12.85%, *P* < 0.001), and specialized epilepsy outpatient clinics of any level were significantly more prevalent among respondents' hospitals in the plains region than in the plateau region (76.0% vs. 55.9%, *P* < 0.001).

**Table 1:** Responses to part I of the questionnaire: characteristics of respondents and their workplace

Question and options	Number (Percentage)		*P*-value
	**Plains group**	**Plateau group**	
1. In what city do you work?	146	179	**-**
2. What type of environment do you work in? (multiple responses possible)			**0.005**^**#**^
Inpatient department in a hospital	136 (93.15)	167 (93.30)	
Outpatient department in a hospital	51 (34.93)	35 (19.55)	
Community clinic	1 (0.68)	8 (4.47)	
Private practice	0	1 (0.56)	
3. What is the level of your workplace?			0.591^#^
Tertiary hospital	133 (91.10)	155 (86.60)	
Secondary hospital	8 (5.48)	17 (9.50)	
Primary hospital	1 (0.68)	2 (1.12)	
Other	4 (2.74)	5 (2.79)	
4. What is your clinical specialty?			** < 0.001**^**#**^
General medicine	8 (5.48)	40 (22.35)	
General internal medicine	22 (15.07)	38 (21.22)	
Neurology	108 (73.97)	23 (12.85)	
Other	8 (5.48)	78 (43.58)	
5. Does your hospital have an epilepsy subspecialty?			0.692^#^
Yes	94 (64.38)	119 (66.48)	
No	52 (35.62)	60 (33.52)	
6. What level of epilepsy unit has been established at your hospital? [30]			0.001^*^
Specialized epilepsy clinic (level I)	74 (50.68)	68 (37.99)	
Epilepsy center (level II)	24 (16.44)	12 (6.70)	
Comprehensive epilepsy center (level III)	13 (8.90)	20 (11.17)	
None	35 (23.97)	79 (44.13)	
7. What is your professional rank?			< 0.001^*^
Resident or junior title	34 (23.29)	93 (51.96)	
Specialist or mid-level title	70 (47.94)	68 (37.99)	
Associate consultant or senior associate title	22 (15.07)	13 (7.26)	
Consultant or senior Positive senior title	20 (13.70)	5 (2.79)	
8. How long have you been working in health care?			0.030^*^
Less than 5 years	19 (13.01)	41 (22.91)	
5–10 years	54 (36.99)	51 (28.49)	
10–15 years	33 (22.60)	51 (28.49)	
More than 15 years	40 (27.40)	36 (20.11)	
9. Please select your gender			0.616^*^
Male	52 (35.62)	59 (32.96)	
Female	94 (64.38)	120 (67.04)	

Data are expressed as Number (Percentage). Bold data show statistical significance at < 0.05

^#^Chi-square test or Fisher's exact test

^*^Mann–Whitney U test (Wilcoxon rank-sum test)

The plains group reported a significantly higher overall score on the questionnaire (85.5 vs. 75.0, *P* < 0.001). Erroneous ideas about the diagnosis of epilepsy were significantly more frequent in the plateau group: higher percentages in that group believed that a first seizure warranted prescription of anti-seizure medication (33.52% vs. 8.22%, *P* < 0.001) and that a first seizure can indicate epilepsy (17.32% vs. 3.42%, *P* < 0.001) (Table 2). Conversely, significantly fewer in the plateau group were aware that electroencephalography (EEG) or computed tomography (CT)/magnetic resonance imaging (MRI) should be carried out when deciding whether initial seizures could be due to epilepsy or another condition (84.36% vs. 95.21%, *P* = 0.002). The plains group also showed significantly greater awareness or knowledge about the "EEG 10–20" system, hyperventilation, flash stimulation, and eye opening/closing during electroencephalographic testing (all *P* < 0.001).

**Table 2:** Responses to part II of the questionnaire: diagnosis of epilepsy

Question and options	Number (Percentage)		*P*-value
	**Plains group** **(*n* = 146)**	**Plateau group** **(*n* = 179)**	
10. Should patients experiencing their first seizures need to be prescribed anti-seizure medications?			** < 0.001**^**#**^
Additional information is needed to answer the question	79 (54.11)	51 (28.49)	
Other response	67 (45.89)	128 (71.51)	
11. Can epilepsy be diagnosed in a patient who has had a single seizure?			**0.009**^**#**^
Additional information is needed to answer the question	88 (60.27)	82 (45.81)	
Other response	58 (39.73)	97(54.19)	
12. What tests need to be prescribed for a first seizure? (multiple responses needed)			**0.002**^**#**^
Electroencephalography and CT/MRI	139 (95.21)	151 (84.36)	
Other response	7 (4.79)	26 (14.53)	
13. Are you aware of the “EEG 10–20” system?			< 0.001^*^
Extremely aware/detailed knowledge	29 (19.86)	15 (8.38)	
Fairly aware/reasonable knowledge	34 (23.29)	16 (8.94)	
Aware/some knowledge	38 (26.03)	54 (30.17)	
Somewhat aware/little knowledge	39 (26.71)	89 (49.72)	
Completely unaware/no knowledge	6 (4.11)	5 (2.79)	
14. Are you aware of the eye-opening and closing response during EEG?			< 0.001^*^
Extremely aware/detailed knowledge	32 (21.91)	17 (9.50)	
Fairly aware/reasonable knowledge	45 (30.82)	20 (11.17)	
Aware/some knowledge	35 (23.97)	65 (36.31)	
Somewhat aware/little knowledge	32 (21.91)	73 (40.78)	
Completely unaware/no knowledge	2 (1.37)	4 (2.23)	
15. Are you aware of hyperventilation as a mode of activation during EEG?			< 0.001^*^
Extremely aware/detailed knowledge	36 (24.66)	16 (8.94)	
Fairly aware/reasonable knowledge	41 (28.08)	21 (11.73)	
Aware/some knowledge	37 (25.34)	65 (36.31)	
Somewhat aware/little knowledge	31 (21.23)	76 (42.46)	
Completely unaware/no knowledge	1 (0.68)	1 (0.56)	
16. Are you aware of flash stimulation as a mode of activation during EEG?			< 0.001^*^
Extremely aware/detailed knowledge	32 (8.22)	16 (8.94)	
Fairly aware/reasonable knowledge	47 (21.91)	21 (11.73)	
Aware/some knowledge	38 (27.40)	64 (35.75)	
Somewhat aware/little knowledge	28 (39.73)	76 (42.46)	
Completely unaware/no knowledge	1 (0.68)	2 (0.56)	

Data are expressed as Number (Percentage). Bold data show statistical significance at < 0.05. *CT *computed tomography, *EEG *electroencephalography, *MRI *magnetic resonance imaging

^#^Chi-squared test or Fisher's exact test

^*^Mann–Whitney U test (Wilcoxon rank-sum test)

Regarding drug-resistant epilepsy, the plains group reported significantly greater awareness or knowledge about its diagnosis and the concept of a defined daily dose for anti-seizure medications (Table 3), which is important because failure to control epilepsy with drugs does not necessarily mean that the condition is drug-resistant; such failure can occur, for example, when drugs are administered at doses below 50% of the defined daily dose [31]. The plains group also showed significantly greater awareness about the usefulness of MRI and single photon emission computed tomography (SPECT) for assessing epilepsy patients before surgery, although the two groups showed equally low awareness of magnetoencephalography (MEG) for this purpose (Table 4). Significantly more plains physicians understood the need for surgery to treat drug-resistant epilepsy involving hippocampal sclerosis or focal cortical dysplasia, and they were more knowledgeable about the indications for preoperative evaluations. Nevertheless, the plains and plateau groups alike showed relatively low knowledge about evaluation based on intracranial electrodes and its indications, as well as about requirements and standardized sequences for MRI of patients with epilepsy. Plains physicians reported significantly more knowledge of deep brain stimulation (DBS) than plateau physicians did, but the two groups reported similar knowledge of VNS, responsive neurostimulation (RNS) and stereoelectroencephalography-guided radiofrequency thermocoagulation (RFTC). The plains group reported significantly more knowledge about MRI-guided laser interstitial thermotherapy (LITT), but the difference from the plateau group seemed confined to physicians who reported knowing relatively little about it (30.17% vs. 19.86%).

**Table 3:** Responses to part III of the questionnaire: drug-resistant epilepsy

Question and options	Number (Percentage)		*P*-value
	**Plains group** **(*n* = 146)**	**Plateau group** **(*n* = 179)**	
17. Are you aware of the criteria for diagnosing drug-resistant epilepsy?			< 0.001^*^
Extremely aware/detailed knowledge	23 (15.75)	16 (8.94)	
Fairly aware/reasonable knowledge	53 (36.30)	27 (15.08)	
Aware/some knowledge	48 (32.87)	64 (35.75)	
Somewhat aware/little knowledge	22 (15.07)	71 (39.67)	
Completely unaware/no knowledge	0	1 (0.56)	
18. Are you aware of the concept of a “defined daily dose” for anti-seizure medications?			0.022^*^
Extremely aware/detailed knowledge	20 (13.70)	17 (9.50)	
Fairly aware/reasonable knowledge	34 (23.29)	21 (11.73)	
Aware/some knowledge	46 (31.51)	73 (40.78)	
Somewhat aware/little knowledge	43 (29.45)	65 (36.31)	
Completely unaware/no knowledge	3 (2.05)	3 (1.68)	

Data are expressed as Number (Percentage). Bold data show statistical significance at < 0.05

^*^Mann–Whitney U test (Wilcoxon rank sum test)

**Table 4:** Responses to part IV of the questionnaire: preoperative evaluation and surgical treatment of drug-resistant epilepsy

Questions and options	Number (Percentage)		*P*-value
	**Plains group** **(*n* = 146)**	**Plateau group** **(*n* = 179)**	
19. Are you aware of the role of pre-MRI in the preoperative evaluation of epilepsy?			0.021^*^
Extremely aware/detailed knowledge	13 (8.90)	12 (6.70)	
Fairly aware/reasonable knowledge	28 (19.18)	16 (8.94)	
Aware/some knowledge	43 (29.45)	55 (30.73)	
Somewhat aware/little knowledge	56 (38.36)	90 (50.28)	
Completely unaware/no knowledge	6 (4.11)	6 (3.35)	
20. Are you aware of the role of SPECT in the preoperative evaluation of epilepsy?			0.036^*^
Extremely aware/detailed knowledge	9 (6.16)	9 (5.03)	
Fairly aware/reasonable knowledge	31 (21.23)	20 (11.17)	
Aware/some knowledge	47 (32.19)	58 (32.40)	
Somewhat aware/little knowledge	52 (35.62)	88 (49.16)	
Completely unaware/no knowledge	7 (4.79)	4 (2.23)	
21. Are you aware of the role of MEG in the preoperative evaluation of epilepsy?			0.881^*^
Extremely aware/detailed knowledge	9 (6.17)	9 (5.03)	
Fairly aware/reasonable knowledge	25 (17.12)	19 (10.61)	
Aware/some knowledge	43 (29.45)	70 (39.11)	
Somewhat aware/little knowledge	62 (42.46)	78 (43.58)	
Completely unaware/no knowledge	7 (4.80)	3 (1.68)	
22. Are you aware that surgery is strongly recommended for drug-resistant epilepsy involving features of hippocampal sclerosis?			< 0.001^*^
Extremely aware/detailed knowledge	20 (13.70)	9 (5.03)	
Fairly aware/reasonable knowledge	35 (23.97)	20 (11.17)	
Aware/some knowledge	42 (28.77)	56 (31.29)	
Somewhat aware/little knowledge	43 (29.45)	89 (49.72)	
Completely unaware/no knowledge	6 (4.11)	5 (2.79)	
23. Are you aware that surgery is strongly recommended for drug-resistant epilepsy involving features of focal cortical dysplasia?			0.001^*^
Extremely aware/detailed knowledge	21 (14.38)	11 (6.15)	
Fairly aware/reasonable knowledge	29 (19.86)	16 (8.94)	
Aware/some knowledge	42 (28.77)	60 (33.52)	
Somewhat aware/little knowledge	49 (33.56)	88 (49.16)	
Completely unaware/no knowledge	5 (3.42)	4 (2.23)	
24. Are you aware of the indications for preoperative evaluation of epilepsy?			0.004^*^
Extremely aware/detailed knowledge	14 (9.59)	11 (6.15)	
Fairly aware/reasonable knowledge	30 (20.55)	21 (11.73)	
Aware/some knowledge	58 (39.73)	68 (37.99)	
Somewhat aware/little knowledge	42 (28.77)	79 (44.13)	
Completely unaware/no knowledge	2 (1.37)	0	
25. Are you aware of intracranial electrode evaluation and its indications for patients with epilepsy?			0.943^*^
Extremely aware/detailed knowledge	11 (7.53)	10 (5.59)	
Fairly aware/reasonable knowledge	19 (13.01)	17 (9.50)	
Aware/some knowledge	39 (26.71)	63 (35.20)	
Somewhat aware/little knowledge	76 (52.06)	87 (48.60)	
Completely unaware/no knowledge	1 (0.69)	2 (1.12)	
26. Are you aware of the minimum requirements from the ILAE for conducting MRI scans of patients with epilepsy?			0.269^*^
Extremely aware/detailed knowledge	12 (8.22)	14 (7.82)	
Fairly aware/reasonable knowledge	32 (21.92)	21 (11.73)	
Aware/some knowledge	40 (27.40)	61 (34.08)	
Somewhat aware/little knowledge	57 (39.04)	82 (45.81)	
Completely unaware/no knowledge	5 (3.42)	1 (0.56)	
27. Are you aware of the standardized sequence of MRI scans from the ILAE for patients with epilepsy?			0.089^*^
Extremely aware/detailed knowledge	13 (8.90)	9 (5.03)	
Fairly aware/reasonable knowledge	24 (16.44)	24 (13.41)	
Aware/some knowledge	46 (31.51)	51 (28.49)	
Somewhat aware/little knowledge	57 (39.04)	92 (51.40)	
Completely unaware/no knowledge	6 (4.11)	3 (1.68)	
28. Do you know about DBS and its indications in patients with epilepsy?			0.005^*^
Extremely aware/detailed knowledge	11 (7.53)	9 (5.03)	
Fairly aware/reasonable knowledge	25 (17.12)	19 (10.61)	
Aware/some knowledge	56 (38.36)	58 (32.40)	
Somewhat aware/little knowledge	53 (36.30)	91 (50.84)	
Completely unaware/no knowledge	1 (0.68)	2 (1.12)	
29. Do you know about VNS and its indications in patients with epilepsy?			0.174^*^
Extremely aware/detailed knowledge	12 (8.22)	9 (5.03)	
Fairly aware/reasonable knowledge	18 (12.33)	19 (10.61)	
Aware/some knowledge	50 (34.25)	58 (32.40)	
Somewhat aware/little knowledge	64 (43.84)	91 (50.84)	
Completely unaware/no knowledge	2 (1.37)	2 (1.12)	
30. Do you know about RNS and its indications in patients with epilepsy?			0.093^*^
Extremely aware/detailed knowledge	12 (8.22)	9 (5.03)	
Fairly aware/reasonable knowledge	18 (12.33)	16 (8.94)	
Aware/some knowledge	50 (34.25)	59 (32.96)	
Somewhat aware/little knowledge	64 (43.84)	92 (51.40)	
Completely unaware/no knowledge	2 (1.37)	3 (1.68)	
31. Do you know about SEEG-guided RFTC for minimally invasive surgery and its indications in patients with epilepsy?			0.186^*^
Extremely aware/detailed knowledge	10 (6.85)	10 (5.59)	
Fairly aware/reasonable knowledge	12 (8.22)	17 (9.50)	
Aware/some knowledge	32 (21.92)	54 (30.17)	
Somewhat aware/little knowledge	88 (60.27)	96 (53.63)	
Completely unaware/no knowledge	4 (2.74)	2 (1.12)	
32. Do you know about MRI-guided LITT intracranial electrode evaluation and its indications in patients with epilepsy?			0.021^*^
Extremely aware/detailed knowledge	8 (5.48)	11 (6.15)	
Fairly aware/reasonable knowledge	9 (6.16)	13 (7.26)	
Aware/some knowledge	29 (19.86)	54 (30.17)	
Somewhat aware/little knowledge	92 (63.01)	98 (54.75)	
Completely unaware/no knowledge	8 (5.48)	3 (1.68)	

Data are expressed as Number (Percentage). Bold data show statistical significance at < 0.05. *pre-MRI *pre-magnetic resonance imaging, *SPECT *single photon emission computed tomography, *MEG* magnetoencephalography, *ILAE* international league against epilepsy, *MRI* magnetic resonance imaging, *DBS* deep brain stimulation, *VNS* vagus nerve stimulation, *RNS* responsive neurostimulation, *SEEG* stereoelectroencephalography, *RFTC* radiofrequency thermocoagulation, *LITT* laser interstitial thermal therapy

^*^Mann–Whitney U test (Wilcoxon rank sum test)

The next part of the questionnaire revealed significant gaps among plateau physicians in their knowledge of status epilepticus (Table 5). Early diagnosis and standardized treatment of status epilepticus can control the condition in over two-thirds of epilepsy patients [24]. Plateau physicians also showed significant gaps in knowledge about comorbidities in epilepsy and their risk of sudden death (Table 6).

**Table 5:** Responses to part V of the questionnaire: status epilepticus

Questions and options	Number (Percentage)		*P*-value
	**Plains group** **(*n* = 146)**	**Plateau group** **(*n* = 179)**	
33. Are you aware of the criteria for diagnosing status epilepticus?			< 0.001^*^
Extremely aware/detailed knowledge	51 (34.93)	25 (13.97)	
Fairly aware/reasonable knowledge	72 (49.32)	45 (25.14)	
Aware/some knowledge	19 (13.01)	77 (43.02)	
Somewhat aware/little knowledge	4 (2.74)	32 (17.88)	
Completely unaware/no knowledge	0	0	
34. Are you aware of the medication process for treating status epilepticus?			< 0.001^*^
Extremely aware/detailed knowledge	42 (28.77)	21 (11.73)	
Fairly aware/reasonable knowledge	62 (42.47)	31 (17.32)	
Aware/some knowledge	35 (23.97)	82 (45.81)	
Somewhat aware/little knowledge	7 (4.79)	45 (25.14)	
Completely unaware/no knowledge	0	0	

Data are expressed as Number (Percentage). Bold data show statistical significance at < 0.05

^*^Mann–Whitney U test (Wilcoxon rank sum test)

**Table 6:** Responses to part VI of the questionnaire: comorbidities and sudden death in epilepsy

Questions and options	Number (Percentage)		*P*-value
	**Plains group** **(*n* = 146)**	**Plateau group** **(*n* = 179)**	
35. Are you aware of the process for assessing epilepsy comorbidities?			0.004^*^
Extremely aware/detailed knowledge	19 (13.01)	11 (6.15)	
Fairly aware/reasonable knowledge	26 (17.81)	21 (11.73)	
Aware/some knowledge	54 (36.99)	68 (37.99)	
Somewhat aware/little knowledge	46 (31.50)	77 (43.02)	
Completely unaware/no knowledge	1 (0.68)	2 (1.12)	
36. Are you aware of SUDEP and its risk factors?			< 0.001^*^
Extremely aware/detailed knowledge	18 (12.33)	11 (6.15)	
Fairly aware/reasonable knowledge	61 (41.78)	34 (18.99)	
Aware/some knowledge	49 (33.56)	77 (43.02)	
Somewhat aware/little knowledge	18 (12.33)	56 (31.28)	
Completely unaware/no knowledge	0	1 (0.56)	

Data are expressed as Number (Percentage). Bold data show statistical significance at < 0.05. *SUDEP* sudden unexpected death in epilepsy

^*^Mann–Whitney U test (Wilcoxon rank sum test)

Finally, plateau physicians showed significant deficits in their knowledge of how to manage epilepsy during pregnancy (Table 7), which may require adjusting the dose of folic acid supplementation, anti-seizure medication and breastfeeding schedule. Pregnancy involves hormonal changes that can alter seizure mechanisms and efficacy of anti-seizure medications, while the drugs can harm the fetus through direct teratogenic effects and through interactions with maternal hormones. Folic acid supplements can help reduce risk of congenital malformations in children born to women on anti-seizure medication [22, 23].

**Table 7:** Responses to part VII of the questionnaire: management of epilepsy in pregnancy

Questions and options	Number (Percentage)		*P*-value
	**Plains group** **(*n* = 146)**	**Plateau group** **(*n* = 179)**	
37. Do you know the recommendations for folic acid supplementation for WWE?			0.002^*^
Extremely aware/detailed knowledge	28 (19.18)	26 (14.53)	
Fairly aware/reasonable knowledge	44 (30.14)	32 (17.88)	
Aware/some knowledge	44 (30.14)	60 (33.52)	
Somewhat aware/little knowledge	27 (18.49)	57 (31.84)	
Completely unaware/no knowledge	3 (2.05)	4 (2.23)	
38. Do you know the process of adjusting antiseizure medications for WWE?			< 0.001^*^
Extremely aware/detailed knowledge	12 (8.22)	10(5.59)	
Fairly aware/reasonable knowledge	61 (41.78)	19(10.61)	
Aware/some knowledge	49 (33.56)	64(35.75)	
Somewhat aware/little knowledge	18 (12.33)	85(47.49)	
Completely unaware/no knowledge	0	1(0.56)	
39. Do you know the postnatal breastfeeding guidelines for WWE?			< 0.001^*^
Extremely aware/detailed knowledge	23 (15.75)	13 (7.26)	
Fairly aware/reasonable knowledge	31 (21.23)	27 (15.08)	
Aware/some knowledge	61 (41.78)	63 (35.20)	
Somewhat aware/little knowledge	29 (19.86)	71 (39.67)	
Completely unaware/no knowledge	2 (1.37)	5 (2.79)	

Data are expressed as Number (Percentage). Bold data show statistical significance at < 0.05. *WWE* women with epilepsy

^*^Mann–Whitney U test (Wilcoxon rank sum test)

We further selected 108 neurologists in the plains region and 23 neurologists in the plateau region to compare their epilepsy knowledge levels (Table 8). The difference in total questionnaire scores between the two groups was not statistically significant (89 vs. 96, *P* = 0.728), and the scores for each module also showed no significant differences (*P* > 0.05 for all modules).

**Table 8:** Comparison of epilepsy awareness scores among psychiatrists in the plains and plateau regions for each module

Epilepsy knowledge module	Median (IQR)		*P*-value
	**Plains group (*N*** **= 108)**	**Plateau group (*N*** **= 23)**	
Diagnosis of epilepsy	19 (16–21.75)	20 (14–22)	0.829^*^
Drug-resistant epilepsy	7 (6–8)	7 (6–9)	0.588^*^
Preoperative evaluation and surgical treatment of drug-resistant epilepsy	40 (32–48)	43 (30–51)	0.744^*^
Status epilepticus	8 (8–10)	8 (8–10)	0.542^*^
Comorbidities of epilepsy and SUDEP assessment	7 (6–8)	7 (6–8)	0.636^*^
Management of epilepsy in pregnancy	10 (8–12)	11 (9–12)	0.738^*^
Total Scores	89 (79–105.75)	96 (74–110)	0.728^*^

Data are expressed as Median (IQR). *P*-values show statistical significance at < 0.05

^*^Mann–Whitney U test (Wilcoxon rank-sum test)

## Discussion

Our study, which appears to be the first parallel comparison of the relatively more developed plains region and less developed plateau region of western China, characterizes in detail gaps in knowledge or awareness of numerous key aspects of epilepsy care in the plateau region relative to the plains region. Meanwhile, the study also revealed significant gaps in knowledge among physicians in the plains region regarding preoperative evaluation and surgical treatment for epilepsy. The lag in plateau regions may stem from a shortage of specialized neurologists. These issues require urgent resolution through targeted training interventions and appropriate policy development.

Geographical disparities across China in the availability of high-quality knowledge and infrastructure to diagnose, treat and manage epilepsy is a major problem [5, 6, 24, 32–36]. Previous studies have confirmed that differences in disease perceptions due to geographic differences can be ameliorated through education and thus improve patients' quality of life. In Saudi Arabia, although epilepsy is a common disease, it is poorly known by the population in the Asser region [37]. In contrast, this geographic disparity was not evident in the greater Tokyo area and non-urban areas of Japan, possibly due to the more balanced educational system in Japan [38]. Abuawad's study found that clinically trained medical students had a greater understanding of the diagnosis and treatment of epilepsy and were less prejudiced against patients with epilepsy [39]. The study highlights significant disparities in epilepsy knowledge between physicians in the more developed plains region and the less developed plateau region of western China, attributed to the concentration of specialists and resources in the plain and challenges such as poverty and low education levels among patients in the plateau region. Currently, three-quarters of China's epilepsy centers are located in the more developed eastern and western regions [5]. Epilepsy specialists in the Plains have higher titles and are more likely to work in hospitals with specialized epilepsy care centers than physicians in the Plateau region. In contrast, poverty issues and lack of formal education among patients in the Plateau region reduce the likelihood that they will be diagnosed and treated in a hospital [4, 6, 24].

The survey reveals significant deficiencies among physicians in the plateau region and some in the plains region regarding the use of modern preoperative techniques for assessing epilepsy, as well as a limited understanding of epilepsy neuromodulation techniques. Until 2016, China had only 37 specialized epilepsy centers per million patients, mainly concentrated in developed regions. This contrasts with the United States, which had 75 epilepsy specialty centers per million patients in 2019 [40], making it essential to strengthen specialized epilepsy centers in economically underdeveloped regions of China [5].

But it is certain that the deficiencies noted in our survey are much smaller than they were a dozen years ago. Since 2005, epilepsy centers have been strengthened in underserved areas of the country with support from local and national programs [34]. Our sample demonstrated that a relatively large number of primary epileptologists as well as specialists from other disciplines in plateau areas were involved in epilepsy treatment training. Long-term treatment outcomes for epilepsy in rural China also improved significantly, with the proportion of epilepsy patients in rural areas who were seizure-free within one year of anti-seizure medication treatment increasing from approximately 40% in 2012 to approximately 66% in 2020 [41]. In addition, the rate of sudden death among patients with epilepsy in rural areas of western China decreased by 60% between 2010 and 2019 [36]. Although progress has indeed been made in epilepsy care in undeveloped regions of China, our study emphasizes that this development has not benefited all regions equally, and that the diagnosis and long-term treatment of epilepsy in plateau regions continue to require training enhancement.

### Limitations

Our data should be interpreted with caution given that our sample was relatively small and may show recruiting bias because all our participants were physicians who had signed up for epilepsy training courses. The questionnaire lacked participants' age and educational background information, but substituted with more influential factors such as employment duration and specialty practice to assist in the assessment. Inferences regarding differences in epilepsy awareness between physicians in the two regions were primarily based on previous research findings, lacking concrete data support. Comparisons between the plain and plateau groups may introduce confounding factors due to significant differences in the composition of physicians' specialties between these regions. The study further analyzed differences in epilepsy cognition among neurologists in the two regions to help explain the findings. The fact that our questionnaire was cross-sectional prevents us from drawing conclusions about changes in awareness or knowledge of epilepsy care over time as a result of, for example, training or capacity-building.

## Conclusions

Despite progress in addressing geographical healthcare disparities in China, physicians in the less developed western plateau region of the country still show significant gaps in awareness or knowledge about many key aspects of epilepsy care, including diagnosis, drug and surgical treatments, definition of drug-resistant epilepsy, diagnosis and management of status epilepticus, management of comorbidities and complications, and management during pregnancy. These regional disparities are primarily attributed to the insufficient number and low proportion of neurologists with specialized training in plateau areas. Physicians in both plains and plateau regions exhibit a severe lack of awareness regarding advanced techniques for preoperative epilepsy assessment. Targeted training and capacity-building interventions should be implemented to bridge these gaps and deficiencies, with their effectiveness rigorously evaluated through longitudinal studies.

## Supplementary Information

Supplementary Material 1.

## Data Availability

All data generated during this study are included in this article. Further enquiries can be directed to the corresponding author.

## Abbreviations

CT: Computed tomography
DBS: Deep brain stimulation
EEG: Electroencephalography
ILAE: International league against epilepsy
LITT: Laser interstitial thermal therapy
MEG: Magnetoencephalography
MRI: Magnetic resonance imaging
pre-MRI: Pre-magnetic resonance imaging
RFTC: Radiofrequency thermocoagulation
RNS: Responsive neurostimulation
SEEG: Stereoelectroencephalography
SPECT: Single photon emission computed tomography
SUDEP: Sudden unexpected death in epilepsy
VNS: Vagus nerve stimulation
WWE: Women with epilepsy
