# Supplementary material for: Epilepsy awareness among physicians in plains and plateau regions of western China: a cross-sectional study
Source: Acta Epileptol. 2026 Mar 11;8:11. doi: 10.1186/s42494-026-00251-9 (PMC12977748; doi:10.1186/s42494-026-00251-9)
Supplement: Supplementary file 1 — Supplementary Material 1. [file 42494_2026_251_MOESM1_ESM.docx]

**Supplementary Table** Questionnaire to assess awareness of key aspects of epilepsy diagnosis, treatment and management.

| **I. Your clinical practice** | **(9 questions)** |
| --- | --- |
| 1. In what city do you work? | |
| 2. What type of environment do you work in? (e.g. hospital - inpatient, hospital - outpatient, community clinic, private practice) | |
| 3. What is the level of your workplace? (e.g. primary, secondary, tertiary) | |
| 4. What is your clinical specialty? | |
| 5. Does your hospital have an epilepsy subspecialty? | |
| 6. What level of epilepsy unit has been established at your hospital? (based on the categorization of the Chinese Association Against Epilepsy [30]) | |
| 7. What is your professional rank? | |
| 8. How long have you ever been working in health care? | |
| 9. Please select your gender. | |
| **II. Diagnosis of epilepsy** | **(7 questions)** |
| 10. Should patients experiencing their first seizures be prescribed anti-epileptic drugs? | |
| 11. Can epilepsy be diagnosed in a patient who has had a single seizure? | |
| 12. What tests need to be prescribed for a first seizure? | |
| 13. Are you aware of the "electroencephalography 10-20 (EEG 10-20)" system? | |
| 14. Are you aware of the eye-opening and -closing response during electroencephalography (EEG)? | |
| 15. Are you aware of hyperventilation as a mode of activation during EEG? | |
| 16. Are you aware of flash stimulation as a mode of activation during EEG? | |
| **III. Drug-resistant epilepsy** | **(2 questions)** |
| 17. Are you aware of the criteria for diagnosing drug-resistant epilepsy? | |
| 18. Are you aware of the concept of a "defined daily dose" for anti-epileptic drugs? | |
| **IV. Preoperative evaluation and surgical treatment of drug-resistant epilepsy** | **(14 questions)** |
| 19. Are you aware of the role of pre-magnetic resonance imaging (pre-MRI) in the preoperative evaluation of epilepsy? | |
| 20. Are you aware of the role of single photon emission computed tomography (SPECT) in the preoperative evaluation of epilepsy? | |
| 21. Are you aware of the role of magnetoencephalography (MEG) in the preoperative evaluation of epilepsy? | |
| 22. Are you aware that surgery is strongly recommended for drug-resistant epilepsy involving features of hippocampal sclerosis? | |
| 23. Are you aware that surgery is strongly recommended for drug-resistant epilepsy involving features of focal cortical dysplasia? | |
| 24. Are you aware of the indications for preoperative evaluation of epilepsy? | |
| 25. Are you aware of intracranial electrode evaluation and its indications for patients with epilepsy? | |
| 26. Are you aware of the minimum requirements from the International League Against Epilepsy (ILAE) for conducting magnetic resonance imaging (MRI) scans of patients with epilepsy? | |
| 27. Are you aware of the standardized sequence of MRI scans from the ILAE for patients with epilepsy? | |
| 28. Do you know about deep brain stimulation (DBS) and its indications in patients with epilepsy? | |
| 29. Do you know about vagus nerve stimulation (VNS) and its indications in patients with epilepsy? | |
| 30. Do you know about responsive neurostimulation (RNS) and its indications in patients with epilepsy? | |
| 31. Do you know about stereoelectroencephalography-guided (SEEG-guided) radiofrequency thermocoagulation (RFTC) for minimally invasive surgery and its indications in patients with epilepsy? | |
| 32. Do you know about MRI-guided laser interstitial thermotherapy (LITT) intracranial electrode evaluation and its indications in patients with epilepsy? | |
| **V. Status epilepticus** | **(2 questions)** |
| 33. Are you aware of the criteria for diagnosing status epilepticus? | |
| 34. Are you aware of the medication process for treating status epilepticus? | |
| **VI. Comorbidities of epilepsy and sudden unexpected death in epilepsy (SUDEP) assessment** | **(2 questions)** |
| 35. Are you aware of the process for assessing epilepsy comorbidities? | |
| 36. Are you aware of SUDEP and its risk factors? | |
| **VII. Management of epilepsy in pregnancy** | **(3 questions)** |
| 37. Do you know the recommendations for folic acid supplementation for women with epilepsy (WWE)? | |
| 38. Do you know the process of adjusting antiseizure medications for WWE? | |
| 39. Do you know the postnatal breastfeeding guidelines for WWE? | |
